# Assessment of change in quality of life, carcinoid syndrome symptoms and healthcare resource utilization in patients with carcinoid syndrome

**DOI:** 10.1186/s12885-019-5459-x

**Published:** 2019-03-28

**Authors:** Daniel M. Halperin, Lynn Huynh, Jennifer L. Beaumont, Beilei Cai, Rachel H. Bhak, Sahil Narkhede, Todor Totev, Mei S. Duh, Maureen P. Neary, David Cella

**Affiliations:** 1 Department of Gastrointestinal Medical Oncology, The University of Texas MD Anderson Cancer Center, 1515 Holcombe Blvd., Houston, TX 77030 USA; 2 Analysis Group, Inc., 111 Huntington Ave. 14th Floor, Boston, MA 02199 USA; 3 Terasaki Research Institute, 1018 Westwood Blvd., Los Angeles, CA 90024 USA; 4 Department of Medical Social Sciences, Northwestern University Feinberg School of Medicine, 633 North Saint Clair St.19th Floor, Chicago, IL 60611 USA; 5 Novartis Pharmaceuticals Corporation, One Health Plaza, 345/5120C, East Hanover, NJ 07936-1080 USA

**Keywords:** (Limit = 3 to 10): carcinoid syndrome, Somatostatin analogs, Quality of life, FACT-G, Healthcare resource utilization

## Abstract

**Background:**

There is limited information on changes over time in carcinoid syndrome (CS) symptoms and quality of life (QoL). This study assessed change in CS symptoms and QoL in patients treated with somatostatin analogs (SSAs) using the Functional Assessment of Cancer Therapy-General (FACT-G) and Patient-Reported Outcomes Measurement Information System (PROMIS)-29 instruments.

**Methods:**

Patients ≥18 years old with CS symptoms and treated with SSA or non-SSA agents in the United States were recruited through a patient advocacy group to complete a two-part, anonymous online survey. Time point (T) 1 survey was fielded from July–October 2016, and T2 survey followed 6 months later. Clinical characteristics and SSA treatment duration were assessed at T1. FACT-G and PROMIS-29 QoL surveys were administered and CS symptoms were assessed at T1 and T2; proportions of patients not experiencing symptoms were compared by McNemar’s test. Healthcare resource utilization (HRU) was assessed for the T1-T2 interval, and mean difference in QoL score from T1 to T2 by SSA duration was calculated.

**Results:**

Of 117 participants at T1, 89 (76%) completed the T2 survey and served as the study sample; 11 (13%) were treated with SSAs for > 0–2 years, 37 (42%) for > 2–5 years, and 39 (45%) for > 5 years. A higher proportion of patients at T2 vs. T1 reported the following symptoms as not applicable: diarrhea (16% vs. 7%, *p* < 0.05), flushing (28% vs. 18%, p < 0.05), wheezing (78% vs 66%, *p* = 0.008). Most patients (89%) had a physical exam and a mean of 7.2 healthcare provider visits between T1 and T2. Patients treated with SSAs for ≤2 years had a mean positive change of 3.7 in their FACT-G total score between surveys, and 6.0 in an additional set of CS-specific questions. Patients receiving SSAs for > 2 years did not appear to associate with a clinically meaningful improvement in QoL score as assessed by FACT-G between T1 and T2; patients also had no clinically meaningful improvement as assessed by PROMIS-29.

**Conclusions:**

There may be clinically important improvement in QoL as measured by FACT-G in patients in earlier years of receiving SSA, which may not appear in later years of SSA treatment.

**Electronic supplementary material:**

The online version of this article (10.1186/s12885-019-5459-x) contains supplementary material, which is available to authorized users.

## Background

Carcinoid syndrome (CS) results from the secretion of bioactive amines, peptides, and polypeptides by functional neuroendocrine tumors (NETs). Symptoms may include diarrhea, flushing, wheezing, and less frequently carcinoid heart disease, characterized pathologically by predominantly right-sided cardiac valvular fibrosis and clinically by cyanosis and/or peripheral edema [1]. Among patients in the United States (US) with NET identified between 2000 and 2011 in Surveillance, Epidemiology, and End Results (SEER) data of the National Cancer Institute, 19% were found to have CS at diagnosis; some patient subgroups, such as those with metastatic well-differentiated small bowel NET, had CS in over 50% of cases [2].

The first-line systemic therapy for metastatic NETs frequently includes somatostatin analogs (SSAs) such as octreotide or lanreotide [3]. Octreotide, approved in the US in 1988, is indicated for the symptomatic treatment of metastatic carcinoid tumors, targeting severe diarrhea and flushing [4]; lanreotide was approved in 2014 for the treatment of patients with unresectable, well- or moderately-differentiated, locally advanced or metastatic gastroenteropancreatic NET to improve progression-free survival [5] and later in 2017 for CS control [6]. SSAs inhibit the secretion of gastrointestinal hormones and alleviate symptoms of CS associated with advanced NETs, such as diarrhea and flushing, and hormonal syndrome [3].

Patients with NET and CS often report reduced quality of life (QoL), particularly involving fatigue, general health, and physical role limitations. Frequent bowel movements and flushing episodes have been shown to be significantly associated with decreased health-related QoL [7]. While there are several published cross-sectional studies on CS symptoms and QoL, published studies with repeated assessment of CS symptoms and QoL among patients in real-world clinical practice who are frequently treated with SSAs are limited; these studies can provide insight into how treatment impacts CS symptoms and QoL over time [7–9]. The objectives of this prospective, two time point study were to examine change in CS symptoms, QoL, and healthcare resource utilization (HRU) in CS patients using the validated Functional Assessment of Cancer Therapy - General (FACT-G) and Patient-Reported Outcomes Measurement Information System (PROMIS)-29 instruments. As FACT-G does not have a specific subscale assessing QoL related to CS concerns, a new CS-specific subscale was developed using pre-existing validated FACIT questions, and change in QoL from this 29 item questionnaire was also evaluated.

## Methods

### Data source & eligibility

Patients with CS symptoms in the US were recruited via the Neuroendocrine Cancer Awareness Network (NCAN). NCAN is a non-profit patient advocacy group dedicated to raising awareness of neuroendocrine cancer, providing support for caregivers and people with NETs, and funding for NET cancer research. NCAN recruited members to participate in an online, two-part, anonymous survey via newsletter, email, and social media outlets. No patient-identifying information was provided in the responses that were received and analyzed. Eligible patients were at least 18 years old, self-reported a physician diagnosis of NET and CS, and received either SSA or non-SSA treatment for CS symptom control. Recruitment and the baseline survey (time point 1 [T1]) were conducted July–October 2016, and time point 2 (T2) survey was administered approximately 6 months later in January–April 2017.

The survey consisted of demographic characteristics (e.g., gender, age, race) and clinical characteristics (e.g., site of NET, time since NET and CS diagnoses) measured at T1. Questions regarding QoL, treatments received, and CS symptom and severity were administered at T1 and T2. HRU was assessed at T2 for the time period between T1 and T2**.** QoL was assessed using the PROMIS-29 and FACT-G instruments. To assess NET-specific QoL, additional FACT questions were selected from an item library of over 700 existing and tested FACIT items. The questions were selected by the study team based on the scientific literature and review by clinical investigators. These additional questions for the new CS-specific subscale focused on diarrhea, flushing, rash, and cognitive ability. Severity ratings of CS symptoms in the past month were classified as mild, moderate, severe, or not applicable (indicating lack of the symptoms).

Data were de-identified and complied following the patient confidentiality requirements of the Health Insurance Portability and Accountability Act (HIPAA). All study materials were approved by the New England Independent Review Board on May 5, 2016 (NEIRB# 16–168). Patients provided their informed consent prior to responding to the survey questions and received a gift card for a nominal amount ($20) as compensation for their time.

### Statistical analyses

Descriptive analyses assessing patient demographic (at T1) and clinical characteristics (at T1 and T2) were performed using means and standard deviations (SDs) for continuous variables and frequencies and proportions for categorical variables. Characteristics of participants who completed the survey only at T1 vs. those who completed the survey at both T1 and T2 were compared to determine whether there were any differentiating factors between the two different groups; this was analyzed using the Wilcoxon rank sum test for continuous variables and the chi square test (or Fisher’s exact test, as appropriate) for categorical variables.

Subsequent analyses to evaluate differences for survey responses between the 2 time points were restricted to participants who completed the surveys at both T1 and T2. QoL measures were analyzed at T1 and T2. Using data collected from the PROMIS-29 instrument, PROMIS-29 domain (Physical Function, Ability to Participate in Social Roles and Activities, Anxiety, Depression, Fatigue, Sleep Disturbance, and Pain Interference) T-scores were calculated according to instructions from the PROMIS profile scoring manual. This involves rescaling of the raw score into a standardized score with a mean of 50 and a standard deviation (SD) of 10, where a score of 50 is the average for the US general population. A higher T-score represents more of the concept being measured, so it implies a better QoL for positively worded domains and worse QoL for negatively worded domains. For data collected from the FACT instrument, FACT-G item subscale (Physical Well-Being [PWB], Social Well-Being [SWB], Emotional Well-Being [EWB], and Functional Well-Being [FWB]) scores were calculated based on summing the values associated with patient responses where higher values were associated with a higher quality of the concept being measured. Total scores were calculated by summing subscales such that total scores could range from 0 to 108 with a higher score having more favorable QoL [10]. Negatively-phrased questions were reversed, so that a higher score on all FACT scales indicates better QoL.

Using data from T1 and T2, participants were categorized according to whether they showed symptom improvement or worsening in flushing, diarrhea, and wheezing. Participants were classified as improved if they had a reduction of select CS symptoms at T2 (e.g., selected 1 flushing episode per day at T2 vs. 2–3 flushing episodes at T1) and were classified as worsened if they reported more of a select CS symptom at T2. Participants who selected a severity rating of ‘not applicable’ were considered to have the lowest severity rating as it was interpreted that they did not experience the select CS symptom at T2. Responses were categorized from most to least severe per the following: severe > moderate > mild > not applicable and were compared using McNemar’s test, as the data were paired within-patient from T1 and T2.

Analysis of change in QoL scores from T1 to T2 for FACT-G and PROMIS-29 was performed overall, by whether participants’ CS symptoms improved or worsened between T1 and T2 (for FACT-G), and by SSA treatment duration reported at T1. SSA treatment duration was categorized by > 0 to 2 years, > 2 and up to 5 years, and > 5 years; these thresholds were determined based on clinical input that > 0 to 2 years can be regarded as the early stage of SSA treatment. For the overall analysis of change in PROMIS-29, FACT-G, and CS-specific additional FACT questions, and for the analysis of change in FACT-G by symptom change, mean and standard deviation of difference were reported and comparisons of scores between two time points were made using the Wilcoxon signed rank test. For comparing QoL at T1 vs. T2 by SSA duration, the mean difference was calculated, and compared to thresholds for a clinically important difference. A clinical minimally important difference (MID) threshold, which is defined as the smallest difference in score in the domain of interest which patients perceive as beneficial [11], of a mean difference of 3–7 points based on an anchor-based approach for total FACT-G was used. A distribution-based approach was used to determine the MID threshold of > 1/3 of the standard deviation at baseline for the sum of the CS-specific additional FACT items and for PROMIS-29 [12–15]. Anchor-based MIDs have the advantage of mapping score differences to differences in clinical measures whereas distribution-based MIDs are based on the statistical properties of the scale; however, not all QoL instruments have published anchor-based MIDs [13].

HRU between T1 and T2 was described by the number and proportion of participants who had a physical exam, mean number of healthcare provider visits and mean number of hospital visits.

Statistically significant associations were noted for *p* < 0.05. All analyses were conducted using SAS version 9.4.

### Availability of data and materials

The patient-level data generated and analyzed in this study are not publicly available as patient participants provided consent to participate in the survey and were told their survey responses would be reported and published only in summary form.

## Results

### Demographic and clinical characteristics

Among the 117 participants at T1, 89 (76%) also completed the survey at T2 and served as the sample size for the analysis for this study; there were no statistically significant differences between participants who only completed the survey at T1 vs. those who completed surveys at T1 and T2 in either demographic characteristics or CS symptoms experienced (Table 1).

**Table 1:** Comparison of Demographic and Clinical Characteristics between Participants with Carcinoid Syndrome Responding to Survey at Time Point 1 vs. both Time Point 1 and Time Point 2

	All Patients				Only Time Point 1		Both Time Point 1 and Time Point 2			p-value^a^
		(*N* = 117)			(*N* = 28)			(*N* = 89)		
Demographic characteristics										
Male, N (%)	27	(23)		5	(18)		22	(25)		0.452
Age (years), mean [median] (SD)	58.0	[57.0]	(9.8)	54.1	[56.5]	(11.1)	59.2	[59.0]	(9.1)	0.099
Age group, N (%)										0.153
18–34 years	3	(3)		2	(7)		1	(1)		
35–44 years	5	(4)		2	(7)		3	(3)		
45–54 years	31	(26)		6	(21)		25	(28)		
55–64 years	46	(39)		14	(50)		32	(36)		
65–74 years	27	(23)		4	(14)		23	(26)		
75+ years	5	(4)		0	(0)		5	(6)		
Race, N (%)^b^										
Caucasian	106	(91)		23	(82)		83	(93)		0.130
Black or African American	6	(5)		3	(11)		3	(3)		0.148
Hispanic or Latino	5	(4)		2	(7)		3	(3)		0.592
Asian/Pacific Islander	0	(0)		0	(0)		0	(0)		–
Native American/American Indian	1	(1)		1	(4)		0	(0)		0.239
Other	3	(3)		2	(7)		1	(1)		0.142
Region of residence, N (%)										0.586
Northeast	21	(18)		4	(14)		17	(19)		
South	41	(35)		11	(39)		30	(34)		
Midwest	30	(26)		9	(32)		21	(24)		
West	25	(21)		4	(14)		21	(24)		
Clinical characteristics										
Primary site of NET, N (%)^b^										
Lung	11	(9)		0	(0)		11	(12)		0.064
Stomach	11	(9)		5	(18)		6	(7)		0.130
Duodenum	10	(8)		1	(4)		9	(10)		0.448
Jejunum	6	(5)		0	(0)		6	(7)		0.333
Ileum	55	(47)		14	(50)		41	(46)		0.716
Appendix	12	(10)		4	(14)		8	(9)		0.478
Colon	10	(8)		2	(7)		8	(9)		1.000
Rectum	0	(0)		0	(0)		0	(0)		–
Other primary site^c^	30	(26)		5	(18)		25	(28)		0.279
Time since NET diagnosis (years), mean [median] (SD)	8.3	[6.7]	(6.0)	7.5	[6.6]	(5.8)	8.6	[6.8]	(6.0)	0.338
Time since CS diagnosis (years), mean [median] (SD)	7.1	[4.8]	(5.5)	6.1	[4.3]	(5.5)	7.4	[5.1]	(5.6)	0.220
CS symptoms experienced after NET diagnosis, N (%)										
Cyanosis	14	(12)		5	(18)		9	(10)		0.318
Carcinoid diarrhea	114	(97)		28	(100)		86	(97)		1.000
Carcinoid heart	15	(13)		3	(11)		12	(14)		1.000
Flushing	106	(91)		26	(93)		80	(90)		1.000
Peripheral edema	54	(46)		15	(54)		39	(44)		0.367
Wheezing	48	(41)		11	(39)		37	(4)		0.830
Current activity level, N (%)										0.285
I have normal activity, without symptoms	11	(9)		5	(18)		6	(7)		
Have symptoms, but do not require bed rest during waking day	70	(60)		14	(50)		56	(63)		
Require bed rest during < 50% of waking day	30	(26)		8	(29)		22	(25)		
Require bed rest during 50% + of waking day	6	(5)		1	(4)		5	(6)		
Unable to get out of bed	0	(0)		0	(0)		0	(0)		

*Abbreviations*: *NET* neuroendocrine tumor, *SD* standard deviation; *T1* time point 1, *T2* time point 2

^a^*p*-values were calculated to compare characteristics between patients completing survey at T1 only vs. those completing survey at T1 and T2

^b^Respondents were allowed to select multiple responses, so counts and percentages may not sum to the total N or 100%

^c^Other primary NET sites include breast, cecum, intestines, liver, mesentery, pancreas, small intestine, ureter, and unknown

Participants who completed both surveys were predominantly female (75%) and Caucasian (93%) with a mean age of 59.2 years. Mean time from diagnosis of NET to T1 and diagnosis of CS to T1 was 8.6 years and 7.4 years, respectively (Table 1). At T1, 87 (98%) of participants reported use of SSAs for CS symptoms at some time, and of the patients that completed both T1 and T2 surveys, 78 participants (88%) reported continuous use of SSA agents between T1 and T2. SSAs included lanreotide, octreotide, and pasireotide. Non-SSAs included cyproheptadine, diphenoxylate-atropine, diphenhydramine, loperamide, ranitidine, and telotristat.

### Change in CS symptoms and QoL between T1 and T2

**Fig. 1:**
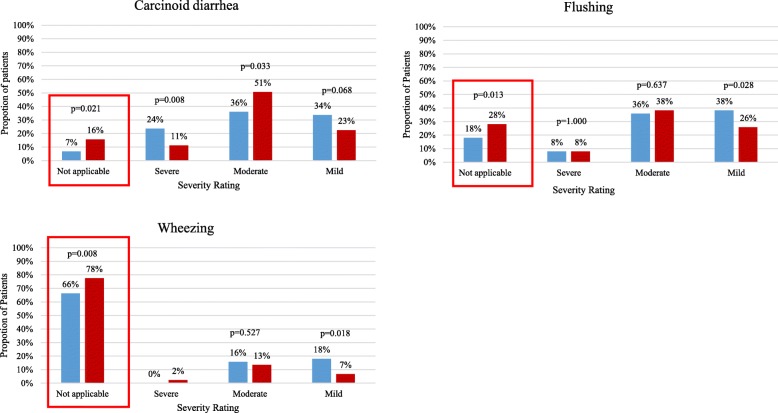
Change in Carcinoid Syndrome Symptoms between Time Point 1 and Time Point 2 for Participants Responding to Survey at both Time Point 1 and Time Point 2.  Time Point 1.  Time Point 2

At T2 compared to T1, a statistically significant higher proportion of patients reported the following CS symptoms were not applicable: carcinoid diarrhea (16% vs 7%, *p* = 0.021), flushing (28% vs 18%, *p* = 0.013), and wheezing (78% vs 66%, *p* = 0.008) (Fig. 1). Participants who had improvement in flushing and diarrhea symptoms between T1 and T2 had a mean improvement of 3.5 in total FACT-G score between T1 and T2, as well as improvement of 11.6 in their CS-specific additional FACT item sum score, both exceeding the MID and showing consistency in the correlation of improved CS symptoms and better QoL. Minimal differences in FACT-G subscales and PROMIS-29 scores between T1 and T2 for those with improvement in flushing and diarrhea symptoms were observed. There was also a statistically significant mean improvement of 2.4 points (*p* = 0.026) in the total score for CS-specific additional FACT-G questions (Additional file 1: Table S1).

Among the 87 patients treated with SSA at T1 or T2, 11 (13%) were treated for > 0 to 2 years, 37 (42%) for > 2 to 5 years, and 39 (45%) for more than 5 years at assessment of SSA duration at T1. Using the FACT-G instrument, an increase in QoL between T1 and T2 was observed in participants in earlier stages of SSA treatment (> 0 to 2 years). Patients who received SSAs for > 0 to 2 years had a mean positive change of 3.7 in their FACT-G total score which is a clinically relevant improvement that exceeds the MID of 3.0 points. Patients who received SSAs for > 2 to 5 years had no change in QoL, and patients who received SSAs for more than 5 years had a decrease of 1.2 in their scores. A borderline clinically meaningful increase of 6.0 (MID = 6.4) in total new CS-specific FACT-G score was found in participants who received SSAs for > 0 to 2 years; increases in scores for patients using SSAs for longer durations were found but were not clinically meaningful (Table 2). There were no clinically meaningful changes observed between T1 and T2 using the PROMIS-29 instrument (Table 3).

**Table 2:** Change in FACT-G Scores between Time Point 1 and Time Point 2 Stratified by SSA Treatment Duration (N = 87)

	Mean Δ FACT-G Scores^a^						Mean Δ CS-Specific Additional FACT Score^a^
	N	Total	Physical well-being	Social well-being	Emotional well-being	Functional well-being	Total
SSA duration at Time Point 1 (years)							
> 0 to 2	11	3.7	1.5	0.1	1.6	0.5	6.0
> 2 to 5	37	0.0	0.6	−0.5	0.1	− 0.1	2.2
> 5	39	−1.2	−0.3	− 0.5	− 0.1	− 0.4	1.9

*Abbreviations*: *FACT-G* Functional Assessment of Cancer Therapy-General, *SSA* somatostatin analog

^a^Change is calculated as the difference in score from Time Point 1 to Time Point 2. A positive change indicates better quality of life

**Table 3:** Change in PROMIS-29 Scores between Time Point 1 and Time Point 2 Stratified by SSA Duration (*N* = 87)

		Mean Δ PROMIS-29 Domain T-scores^a^							
	N	Physical function^b^	Social roles^b^	Anxiety^c^	Depression^c^	Fatigue^c^	Sleep disturbance^c^	Pain interference^c^	Pain intensity^d^
SSA duration at Time Point 1 (years)									
> 0 to 2	11	1.3	0.5	−0.3	0.7	0.7	−0.6	2.6	0.3
> 2 to 5	37	−0.8	0.8	−1.8	0.1	−0.6	−1.5	1.4	−0.1
> 5	39	−0.5	0.2	−0.2	−2.2	0.0	−1.8	1.3	0.4

*Abbreviations*: *PROMIS* Patient-Reported Outcomes Measurement Information System, *SSA*, somatostatin analog

^a^Change is calculated as the difference in scores from Time Point 1 to Time Point 2

^b^A positive change indicates better quality of life

^c^A negative change indicates better quality of life

^d^Pain intensity was not scaled to a T-score. A positive change indicates worse quality of life

### Health resource utilization between T1 and T2

The majority of patients reported having had a physical exam between T1 and T2 (89%). The mean (SD) number of health care provider visits between surveys was 7.2 (13.4), of hospitalizations was 0.3 (0.7), and of number of days of poor health preventing usual activities in the past 30 days was 8.0 (9.5) (Table 4). Additionally, compared to those who did not have improvements in flushing and diarrhea, patients who had improvement in flushing and diarrhea symptoms between T1 and T2 also had a lower mean number of healthcare provider visits (6.38 vs. 7.32) and hospitalizations (0.13 vs. 0.32). Patients treated with SSA for > 0 to 2 years also had fewer mean healthcare provider visits than those treated for > 2 years (5.55 vs. 7.57).

**Table 4:** Health Resource Utilization between Time Point 1 and Time Point 2

	All Time Point 2 Patients
	(*N* = 89)
Health Resource Utilization (Since Time Point 1)	
Had a physical exam, N (%)	79 (89)
Number of health care provider visits, mean [median] (SD)	7.2 [5.0] (13.4)
Number of hospitalizations, mean [median] (SD)	0.3 [0.0] (0.7)
Number of days poor health prevented usual activities in past 30 days, mean [median] (SD)	8.0 [5.0] (9.5)

*Abbreviations*: *SD* Standard Deviation

## Discussion

For this patient population in which a vast majority were treated with SSAs, there was CS symptom improvement for diarrhea, flushing and wheezing over time in terms of a decrease in proportion of patients with symptoms and a decrease in severity of symptoms. Furthermore, these changes in symptoms were associated with improvement of QoL as measured by FACT-G, indicating that general QoL is related to severity of CS symptoms. An improvement in QoL between the two study time points as measured by FACT-G was observed for patients in the earlier years (> 0 to 2 years) of SSA treatment but not for those in later years of SSA treatment.

Patients who were treated with SSAs for longer duration may have been less likely to experience improvement in QoL during the study time period compared with those who initiated treatment more recently for a variety of reasons. SSAs are generally the first prescribed treatment for carcinoid syndrome. It is possible that the clinical benefits of SSAs result in both symptom reduction and improvement in QoL, but these changes are soon after treatment initiation, and additional improvement may not be observed with ongoing treatment. A blinded, placebo-controlled cross-over study demonstrated that octreotide was associated with significant reductions in diarrhea and flushing and improvements in two domains of the Psychosocial Adjustment to Illness Scale (PAIS) over a four week period [16]. Similarly, the current study demonstrates QoL improvements in the short term following initiation of SSAs; there are no long term trial results to compare the current study results for assessing QoL with longer duration of SSA treatment. It is also plausible that patients who were treated for longer in the current study experienced disease progression and late effects of cancer treatment which may have also affected observed changes in QoL. The double-blinded, placebo-controlled study of octreotide long-acting among treatment-naïve patients with metastatic midgut NETs reported that median time to progression was 14.3 months for patients treated with octreotide [17]. In the double-blinded, placebo-control study of lanreotide among patients with enteropancreatic NETs, 48% of patients randomized to lanreotide were alive and had experienced disease progression at week 96. It is possible that patients in the current study who were treated with SSAs for more than two years had progressed with disease and added therapies to manage disease. The progression and other therapies at this stage may have outweighed QoL improvements brought by SSAs. SSAs are known to have a positive effect on QoL due to effectiveness and favorable toxicity profile, but the therapeutic window can be more narrow when SSAs are combined with interferon, for example [18]. Arnold et al. [19] showed lower QoL scores among patients treated with interferon plus octreotide vs. those treated with octreotide monotherapy. Such studies may in part explain why those with longer SSA duration in the current study did not show improved QoL as they may be receiving additional therapies in the later disease states. Adding therapies at that stage would be in agreement with treatment guidelines which advise that other treatments such as everolimus be used after progression on SSAs in GI NETs.

While there are no other real world studies that measure CS symptoms and QoL over time for a population of NET patients treated for CS symptoms, the published literature on cross-sectional studies of CS symptoms and QoL have shown associations between symptom burden and decreased QoL [7, 20]. Even incremental benefits gained in controlling CS symptoms can lead to improvement in QoL. Patients who have as few as one to three episodes of flushing per week reported lower QoL relative to those with no episodes in one cross-sectional study [7]. A small crossover trial demonstrated SSAs relieve symptoms among patients with GEP-NETs in parallel with QoL improvement, demonstrating symptom control was essential to restoring QoL. Given the observed improvement in QoL among those treated with SSAs in this study, it may also be of interest for future research to assess related drug costs to further contextualize the benefit conferred.

In the current study, meaningful improvements in QoL over time were found among those with shorter SSA treatment duration when the FACT-G instrument was used but not when PROMIS-29 was used. Both the analyses with the FACT-G and new CS-specific subscale showed improvement over time for this treatment group. This may be due to FACT-G’s PWB and FWB subscales containing disease- and treatment-specific items whereas PROMIS-29’s general QoL attributes were designed for a wide range of chronic diseases. Assessment of the CS-specific additional FACT sum score was a unique way in which this study could evaluate QoL for qualities relevant to this specific patient population.

QoL assessments provide important information on treatments as they represent the patient’s direct perspective [18]. QoL measures capture information on how patients live which are not captured by measures of prolongation of life often reported in clinical research [21]. Importantly, self-report of a patient’s health status is without interpretation by a third person [22]. For illnesses such as NET which may be indolent or slowly progressive, understanding QoL and how it changes with treatment may be an important factor in determining treatment options [23].

The current study sought to complement existing literature by overcoming some limitations of prior NET-related QoL studies [22]. The current study had data from two time points rather than being cross-sectional, and it assessed a range of health issues beyond physical abilities. This survey is the first known study to assess QoL across time points, and it had high recapture yield of patients at the second time point. The survey was administered online with a finite set of response options which helped reduce missing data. Nonetheless, there are some limitations of the current study. First, recruitment was conducted primarily through NCAN, which may have resulted in a potentially biased sample not fully representative of the heterogeneous NET patient population; these patients may be more engaged and likely to seek care than other patients with similar disease. Second, the sample of patients was heterogeneous with respect to disease characteristics; there was no ability to look at clinical information such as tumor burden or biochemical markers to assess associations between these factors and QoL. Third, there was no distinction between types of SSAs such as long-acting and short-acting; however, prior studies have shown no difference in efficacy for controlling CS for these SSAs [24]. Additionally, CS treatment was not examined at the agent specific level, whether SSA (e.g., octreotide) or non-SSA (e.g., telotristat), or by continuous or intermittent use. Further research could assess whether differences in these study outcomes exist at the specific agent or continuous use level. Fourth, as with any survey, potential responder bias may exist. Eighty nine of 117 (76%) respondents at T1 completed the survey at T2. There may be differences between those who participate in one vs. both survey rounds, however, a comparison of demographic and clinical characteristics at T1 of these two groups of responders showed no differences in these measureable characteristics. Additionally, all data were self-reported and could have been subject to recall bias. In addition, reporting of symptoms were identified as CS-related, but causes of the symptom (e.g., treatment-related side effects) were not determined. Finally, the sample size was somewhat limited, especially given the breakdown by SSA duration and the findings for those treated with SSAs for > 0 to 2 years. Therefore, the observed improvement in QoL should be validated through further research and a more robust sample size.

## Conclusions

This survey conducted at two time points suggested there may be clinically important improvement in QoL as measured by FACT-G in patients treated with SSAs, which may not appear in later years of SSA treatment.

## Additional file

Additional file 1: **Table S1.** Change in Quality of Life Scores between Time Point 1 and Time Point 2: New CS-specific FACT-G Subscale. **Table S2.** Change in Quality of Life Scores between Time Point 1 and Time Point 2 for Participants with Carcinoid Syndrome: PROMIS-29 and FACT-G. (DOCX 28 kb)
