# Supplementary material for: Assessment of change in quality of life, carcinoid syndrome symptoms and healthcare resource utilization in patients with carcinoid syndrome
Source: BMC Cancer. 2019 Mar 28;19:274. doi: 10.1186/s12885-019-5459-x (PMC6437890; doi:10.1186/s12885-019-5459-x)
Supplement: Supplementary file 1 — Table S1. Change in Quality of Life Scores between Time Point 1 and Time Point 2: New CS-specific FACT-G Subscale. Table S2. Change in Quality of Life Scores between Time Point 1 and Time Point 2 for Participants with Carcinoid Syndrome: PROMIS-29 and FACT-G. (DOCX 28 kb) [file 12885_2019_5459_MOESM1_ESM.docx]

#### Table S1. Change in Quality of Life Scores between Time Point 1 and Time Point 2: New CS-specific FACT-G Subscale

|  |  |  |  |  |  |  |  |
| --- | --- | --- | --- | --- | --- | --- | --- |
|  | Patients who completed both Time Point 1 and Time Point 2 | | | | | | |
|  | (N=89) | | | | | | |
|  | At Time Point 1 | | At Time Point 2^1^ | | QoL score change | | p-value^1^ |
| **Additional FACT question scores^2^, mean (SD)** |  |  |  |  |  |  |  |
| I have flushing episodes | 2.4 | (1.2) | 2.4 | (1.2) | 0.0 | (0.9) | 0.908 |
| I am bothered by flushing episodes | 2.7 | (1.2) | 2.8 | (1.3) | 0.1 | (1.0) | 0.264 |
| I have diarrhea | 1.7 | (1.0) | 1.8 | (1.0) | 0.1 | (0.9) | 0.367 |
| My problem with diarrhea keeps/wakes me up at night | 2.9 | (1.2) | 3.1 | (1.1) | 0.2 | (1.0) | 0.076 |
| I am afraid to be far from a toilet | 2.2 | (1.3) | 2.1 | (1.3) | 0.0 | (0.9) | 0.847 |
| I have to limit my activities due to diarrhea | 2.3 | (1.3) | 2.4 | (1.2) | 0.2 | (0.9) | 0.049* |
| I have to limit my social activity because of diarrhea | 2.3 | (1.3) | 2.5 | (1.2) | 0.1 | (0.9) | 0.089 |
| I have to take my condition into account when making plans | 1.5 | (1.3) | 1.6 | (1.2) | 0.1 | (0.9) | 0.249 |
| I have discomfort or pain in my stomach area | 2.2 | (1.3) | 2.2 | (1.4) | 0.0 | (1.0) | 0.857 |
| I have abdominal cramps or discomfort (due to my diarrhea) | 2.2 | (1.2) | 2.2 | (1.3) | -0.1 | (1.1) | 0.544 |
| I feel bloated | 2.0 | (1.2) | 2.1 | (1.3) | 0.1 | (1.0) | 0.307 |
| I have been short of breath | 2.5 | (1.3) | 2.5 | (1.3) | 0.0 | (1.0) | 0.901 |
| At times I feel breathless | 2.8 | (1.3) | 2.7 | (1.3) | -0.1 | (1.1) | 0.450 |
| I have a rash or skin reddening on my face, neck or chest | 3.1 | (1.3) | 3.0 | (1.3) | -0.1 | (1.0) | 0.382 |
| I am bothered by a skin rash | 3.3 | (1.1) | 3.4 | (1.0) | 0.0 | (1.0) | 0.703 |
| I have hot flushes | 2.5 | (1.2) | 2.6 | (1.2) | 0.1 | (1.2) | 0.527 |
| I am able to eat the foods that I like^3^ | 1.9 | (1.0) | 2.1 | (1.0) | 0.2 | (1.2) | 0.034* |
| I am bothered by a change in weight | 2.6 | (1.2) | 2.5 | (1.5) | 0.0 | (1.2) | 0.894 |
| I worry about the effect of stress on my illness | 2.0 | (1.2) | 2.1 | (1.2) | 0.2 | (1.0) | 0.175 |
| Certain foods or drinks can make my symptoms worse | 1.2 | (1.1) | 1.5 | (1.2) | 0.3 | (0.9) | 0.001* |
| Physical or emotional stress can make my symptoms worse | 1.2 | (1.1) | 1.5 | (1.3) | 0.3 | (1.0) | 0.003* |
| I am able to do my usual activities^3^ | 2.0 | (1.2) | 2.0 | (1.2) | 0.0 | (1.0) | 0.773 |
| I am bothered by itching | 2.9 | (1.4) | 2.9 | (1.3) | 0.0 | (1.1) | 0.784 |
| I have had side-effects from my treatment | 2.6 | (1.2) | 2.8 | (1.1) | 0.2 | (1.1) | 0.157 |
| I have had a problem from repeated injections | 3.2 | (1.1) | 3.2 | (1.1) | 0.0 | (1.1) | 0.951 |
| Subtotal^4^ | 58.0 | (16.3) | 60.0 | (16.5) | 2.0 | (9.4) | 0.035* |
| I have had trouble concentrating | 2.0 | (1.3) | 2.0 | (1.2) | 0.0 | (0.9) | 0.561 |
| My thinking has been slow | 2.1 | (1.3) | 2.1 | (1.2) | 0.0 | (1.0) | 0.742 |
| I have trouble remembering information | 1.8 | (1.2) | 2.0 | (1.1) | 0.2 | (0.9) | 0.039* |
| I have trouble thinking clearly | 2.2 | (1.4) | 2.4 | (1.2) | 0.1 | (1.0) | 0.282 |
| Subtotal^4^ | 8.1 | (5.0) | 8.5 | (4.4) | 0.4 | (3.3) | 0.326 |
| **Total score^4^, mean (SD)** | 66.1 | (19.3) | 68.5 | (19.2) | 2.4 | (10.5) | 0.026* |
| ^1^p-values were calculated to compare QoL at Time Point 1 vs. Time Point 2. Asterisk (*) indicates p-value<0.05. | | | | | | | |
| ^2^Higher score indicates better quality of life. |  |  |  |  |  |  |  |
| ^3^Question reverse-scored for contextual consistency with the rest of the attributes. | |  |  |  |  |  |  |
| ^4^Subtotal scores were calculated as the sum of the scores for the 25 and 4 attributes. The possible ranges were 0-100 and 0-16, respectively. Total score was calculated as the sum of the scores across all 29 attributes with a possible range was from 0-116. | | | | | | | |

#### Table S2. Change in Quality of Life Scores between Time Point 1 and Time Point 2 for Participants with Carcinoid Syndrome: PROMIS-29 and FACT-G

|  |  | Participants who completed surveys at both Time Point 1 and Time Point 2 | | | | | | | | | | |  |
| --- | --- | --- | --- | --- | --- | --- | --- | --- | --- | --- | --- | --- | --- |
|  |  | (N=89) | | | | | | | | | | |  |
|  | Possible range | At Time Point 1 | |  | At Time Point 2 | |  | QoL score change | |  | p-value^1^ | |  |
| **PROMIS-29 scores** |  |  | | | | | | | |  |  | |  |
| PROMIS-29 domain T-score, mean (SD) |  |  |  |  |  |  |  |  |  |  |  | |  |
| Physical function^2^ | 22.9 - 56.9 | 42.7 | (8.1) |  | 42.4 | (8.3) |  | -0.3 | (4.7) |  | 0.360 | |  |
| Ability to participate in social roles and  Activities^2^ | 27.5 - 64.2 | 44.5 | (8.1) |  | 44.9 | (9.0) |  | 0.4 | (5.2) |  | 0.405 | |  |
| Anxiety^3^ | 40.3 - 81.6 | 55.4 | (9.8) |  | 54.5 | (9.3) |  | -0.9 | (6.6) |  | 0.247 | |  |
| Depression^3^ | 41.0 - 79.4 | 52.8 | (8.7) |  | 52.0 | (8.8) |  | -0.8 | (7.9) |  | 0.551 | |  |
| Fatigue^3^ | 33.7 - 75.8 | 59.0 | (9.6) |  | 58.9 | (10.0) |  | -0.1 | (6.9) |  | 0.788 | |  |
| Sleep disturbance^3^ | 32.0 - 73.3 | 53.7 | (7.8) |  | 52.2 | (7.3) |  | -1.5 | (5.3) |  | 0.009* | |  |
| Pain interference^3^ | 41.6 - 75.6 | 53.5 | (10.1) |  | 55.1 | (10.4) |  | 1.6 | (5.9) |  | 0.015* | |  |
| Pain intensity, mean (SD) | 0 - 10 | 3.1 | (2.7) |  | 3.3 | (2.5) |  | 0.2 | (1.5) |  | 0.144 | |  |
| **FACT-G scores, mean (SD)** |  |  | | | | | | | |  |  | |  |
| FACT-G total | 0 - 108 | 67.9 | (19.3) |  | 67.6 | (20.3) |  | -0.3 | (9.6) |  | 0.523 | |  |
| Physical well-being^2^ | 0 - 28 | 18.2 | (6.2) |  | 18.4 | (6.3) |  | 0.3 | (3.6) |  | 0.420 | |  |
| Social well-being^2^ | 0 - 28 | 18.4 | (6.6) |  | 17.9 | (6.6) |  | -0.5 | (3.3) |  | 0.156 | |  |
| Emotional well-being^2^ | 0 - 24 | 16.1 | (4.5) |  | 16.3 | (4.8) |  | 0.2 | (3.1) |  | 0.746 | |  |
| Functional well-being^2^ | 0 - 28 | 15.2 | (6.6) |  | 14.9 | (6.8) |  | -0.3 | (3.7) |  | 0.305 | |  |
| Abbreviations: FACT-G, Functional Assessment of Cancer Therapy-General; PROMIS, Patient-Reported Outcomes Measurement Information System; QoL, quality of life; SD, standard deviation. ; *, p-value<0.05.  ^1^p-values were calculated to compare QoL at Time Point 1 vs. Time Point 2. Asterisk (*) indicates p-value<0.05.  ^2^Higher score indicates better quality of life.  ^3^Higher score indicates worse quality of life. | | | | | | | | | | | |  | |
